# Impact of CD3 expression on outcome in pediatric anaplastic large cell lymphoma

**DOI:** 10.3389/fonc.2025.1569370

**Published:** 2025-05-15

**Authors:** Ahmed Salah, Hanafy Hafez, Samah Semary, Eman Naguib Khorshed, Sonia Soliman, Iman Zaky, Shahenda M. Shahin, Leslie Lehmann, Alaa ElHaddad

**Affiliations:** ^1^ Department of Pediatric Oncology, Children’s Cancer Hospital (57357), Cairo, Egypt; ^2^ Department of Pediatric Oncology, National Cancer Institute, Cairo, Egypt; ^3^ Department of Clinical Oncology, Faculty of Medicine, Beni-Suef University, Beni-Suef, Egypt; ^4^ Department of Surgical Pathology, Children’s Cancer Hospital (57357), Cairo, Egypt; ^5^ Department of Surgical Pathology, National Cancer Institute (NCI), Cairo University, Cairo, Egypt; ^6^ Department of Clinical Pathology, Children’s Cancer Hospital (57357), Cairo, Egypt; ^7^ Department of Clinical Pathology, National Cancer Institute (NCI), Cairo University, Cairo, Egypt; ^8^ Department of Radio-diagnosis, Children’s Cancer Hospital (57357), Cairo, Egypt; ^9^ Department of Clinical Research, Children’s Cancer Hospital (57357), Cairo, Egypt; ^10^ Department of Pediatric Oncology, Dana- Farber Cancer Institute, Boston, MA, United States

**Keywords:** CD3, survival, pediatric anaplastic large cell lymphoma, ALK, outcome

## Abstract

**Background:**

Anaplastic large cell lymphoma (ALCL) constitutes 10-15% of childhood non-Hodgkin lymphoma. EFS is 70% and currently 80% with the additional of targeted agents such as CD30 directed conjugated monoclonal antibody brentuximab or ALK inhibitors such as crizotinib. Expression of CD3, a T-cell marker, can be lost or diminished in some ALCL cases. The literature is conflicting on whether CD3 expression affects prognosis, and it has been analyzed mostly in the relapse setting. The purpose of this study was to determine the effect of CD3 expression on survival and its relation to the other prognostic variables in newly diagnosed patients with pediatric ALCL treated at a single large pediatric oncology center.

**Methods:**

A retrospective study was done on 89 newly diagnosed pediatric ALCL patients (under 18 years old) treated at Children’s Cancer Hospital Egypt (CCHE-57357) from July 2007 to December 2019. Immunohistochemistry was utilized to confirm the diagnosis and determine CD3 expression in tumor cells. The impact of CD3 expression on event-free survival (EFS), relapse-free survival (RFS) and overall survival (OS)) was analyzed.

**Results:**

The median age was 10.7 years with male to female ratio 1.8:1. The majority of patients (85.4%) were ALK positive. CD3 was positive in 31 (34.8%) of patients. The median follow-up period was 60 months. The five-year OS, EFS, and RFS rates for the entire group were 84.3%, 73.1%, and 81.5%, respectively. CD3 positivity was associated with a higher incidence of CNS involvement (p=0.03) but did not significantly impact other patient outcomes (EFS, RFS and OS). However, stage, B symptoms, and skin involvement were linked to a shorter relapse-free survival.

**Conclusion:**

This study indicates that CD3 expression may not be a major factor predicting survival in newly diagnosed pediatric ALCL. Additional research is needed to understand its association with CNS positive disease.

## Introduction

Anaplastic large cell lymphoma (ALCL) accounts for 10% to 15% of all childhood non-Hodgkin lymphomas (1). This tumor is characterized by strong expression of CD30. The majority of pediatric cases also demonstrate overexpression of anaplastic lymphoma kinase protein (ALK) (2).

Since the addition of novel agents to standard chemotherapy has improved outcome. Also targeted monotherapy or combination targeted therapy has shown efficacy in relapsed ALCL. EFS is 70% and currently 80% with the additional of targeted agents such as CD30 directed conjugated monoclonal antibody brentuximab or ALK inhibitors such as crizotinib (2).

CD3 is a T-cell marker that can be detected by immunohistochemistry (IHC) on tissue specimens or flow cytometry on peripheral blood samples (3). It is often expressed in ALCL but in some cases CD3 expression is lost or diminished. It has been postulated that this may have an impact on outcome (4).

The incidence and relevance of CD3 expression in pediatric ALCL are not firmly established. Some studies have found CD3 expression to be more prevalent in ALK-negative versus ALK-positive ALCL which may confound the association with a worse outcome (4).

A recent clinical trial (ALCL-Relapse) investigated the role of stem cell transplantation (SCT) versus vinblastine monotherapy for relapsed or refractory pediatric ALCL. The trial stratified patients based on the time of relapse and CD3 expression to prospectively test different reinduction approaches combined with consolidation via allogeneic or autologous SCT as compared to vinblastine monotherapy (5). The study reported CD3 expression in 28% of patients with ALK-positive ALCL and 67% of patients with ALK-negative ALCL. CD3 expression was associated with a higher risk of relapse after SCT, but not with overall survival. The trial also demonstrated that vinblastine monotherapy was effective and well tolerated as maintenance therapy after SCT (5).

This study’s objectives were to assess the impact of CD3 expression on the outcomes in the setting of newly diagnosed ALCL in the pediatric age group, and evaluate the relationship between CD3 expression and other known prognostic factors

## Patients and methods

This retrospective study comprised 89 newly diagnosed patients under age 18 who were treated at Children’s Cancer Hospital Egypt (CCHE) between July 2007 and December 2019. Patients with primary cutaneous T cell lymphoma, an underlying immunodeficiency or whose paraffin blocks were unavailable were excluded.

Retrospective review of medical records included history, physical examination, full blood count, bone marrow (BM) aspirate and bilateral bone marrow biopsy, and cerebrospinal fluid cytology. Plain X-rays, ultrasonography, computed tomography, PET CT scan (if available), and magnetic resonance imaging were used for radiologic staging. Patients were staged using the Murphy staging system (6), and chemotherapy was administered according to the Children’s Oncology Group ANHL 0131 protocol, Vinblastine arm (7).

Paraffin blocks were collected from the Pathology Department’s archives. The pathologist reviewed H&E stained and immunostained slides to confirm the diagnosis and examine the immunohistochemical expression of CD3, CD30, and ALK in tumor cells.

Immunostaining was performed with the BenchMark Ventana Autostainer. Positive CD3 expression was defined as a membrane/cytoplasmic response in neoplastic cells regardless of the strength of staining or percent of positive cells. Univariant analysis was used to determine the correlation between CD3 expression in ALCL cells and overall (OS), event-free survival (EFS), and clinical characteristics such as age, disease stage, and treatment response.

### Definitions

**Complete remission (CR):** Defined as disappearance of all evidence of disease

**Complete response, unconfirmed (Cru):** Defined as a residual lymph node mass > 1.5 cm that regressed by >75% in the sum of the products of the greatest perpendicular diameters (SPD) or any residual lesions in organs that decreased by >75% with a negative gallium scan and/or PET scan.

**Partial response (PR):** defined as a ≥ 50% decrease in the SPD of the lesions with no new lesion

**Progressive Disease (PD):** defined as the appearance of any new lesion or any evidence of primary tumor progression (6).

### Statistical analysis

We summarized patient characteristics using frequencies and percentages for categorical data and mean (standard deviations) or median (IQR) for continuous variables.

The chi-squared or Fisher’s exact tests were used to examine the relationship between CD3 status and other clinical factors. The EFS and OS were analyzed using Kaplan-Meier, and differences between CD3 expression statuses were examined using the Log-rank test. The Cox regression method was used to determine the difference in survival rates between CD3 status and other confounding variables. All of these data were examined with SPSS version 25, and a 95% confidence interval was employed in all analyses.

## Results

### Patient characteristics

The median age of the 89 patients was 10.7 years (range: 1–18 years), with 58 (65.2%) being male and a male-to-female ratio of 1.8:1. 76 (85.4%) had ALK-positive ALCL and 13 (14.6%) had ALK-negative ALCL. 44 patients (49.4%) were stage III, whereas 17 (19.1%) were stage IV (**Table 1**).

**Table 1:** Patient’s characteristics in relationship to CD3 status.

Freq	Status	Total	CD3 Positive	CD3 negative	P value
Age Med: 10.7 year	>11 year	44	16 (36.4%)	28 (63.6%)	0.76
	<11 year	45	15 (33.3%)	30% (66.7%)	
Sex M:F: 1.8:1	Female	31 (34.8%)	10 (32.3%)	21 (67.7%)	0.70
	Male	58 (65.2%)	21 (36.2%)	37 (63.8%)	
ALK Status	Positive	76 (85.4%)	28 (36.8%)	48 (63.2%)	0.33
	Negative	13 (14.6%)	3 (23.1%)	10 (76.9%)	
Stage	Stage I	7 (7.8%)	2 (28.6%)	5 (67.4)	0.08
	Stage II	21 (22.4%)	4 (19%)	17 (81%)	
	Stage III	44 (49.4%)	15 (34.1)	29 (64.9%)	
	Stage IV	17 (19.1%)	10 (58.8%)	7 (41.2%)	
B Symptoms	Yes	23 (25.8%)	11 (47.8%)	12 (52.2%)	0.12
	No	66 (74.2%)	20 (30.3%)	46 (69.7%)	
BM Involvement	Yes	2 (2.2%)	1 (50%)	1 (50%)	0.64
	No	87 (97.8%)	30 (34.5%)	57 (65.5%)	
Skin Involvement	Yes	9 (10.1%)	2 (22.2%)	7 (77.8%)	0.38
	No	80 (89.9%)	29 (36.2)	51 (63.8%)	
Bone Involvement	Yes	10 (11.2%)	5 (50%)	5 (50%)	0.28
	No	79 (88.8%)	26 (32.9%)	53 (67.1%)	
Visceral Involvement	Yes	9 (10.1%)	2 (22.2%)	7 (77.8%)	0.38
	No	80 (89.9%)	29 (36.3%)	51 (63.8%)	
CNS Involvement	Yes	9 (10.1%)	6 (66.7%)	3 (33.3%)	0.03
	NO	80 (89.9%)	25 (31.3%)	55 (68.7%)	
Response Post Induction	CR	59 (66.3%)	20 (33.9%)	39 (66.1%)	0.97
	CRu	14 (15.7%)	5 (35.7%)	9 (64.3%)	
	Died	3 (3.4%)	1 (33.3%)	2 (66.7%)	
	PD	2 (2.2%)	1 (50%0	1 (50%)	
	PR	10 (11.2%)	4 (40%)	6 (60%)	
	Stable	1 (1.1%)	1 (100%)	0 (0%)	
Time of Relapse	Early*	7 (43.8%)	2 (28.6%)	5 (71.4%)	0.63
	Late*	9 (56.3%)	4 (44.4%)	5 (55.6%)	

*Early Relapse: up to 12 months from initial diagnosis * Late Relapse: More than 12 months from initial diagnosis.

B symptoms were detected in 23 individuals (25.8%) and involvement was assessed in bone marrow (BM), skin, bone, visceral organs, and central nervous system (CNS). Two patients (2.2%) had BM involvement, nine (10.1%) had skin involvement, ten (11.2%) had bone involvement, nine (10.1%) had visceral involvement, and nine (10.1%) had CNS involvement (**Table 1**). A univariate analysis of the association between CD 3 expression and other known prognostic factors was done.

### Survival outcomes

The median follow-up period was 60 months. The five-year OS, EFS, and RFS rates for the entire group were 84.3%, 73.1%, and 81.5%, respectively.

There were no significant differences in OS, EFS, or RFS based on patients’ characteristics (**Table 2**). However, there was a significant difference in RFS based on stage, B symptoms and skin involvement with (**Table 2**).

**Table 2:** Univariant analysis: patient’s characteristics and survival outcome.

Freq	Status	Total	OS	P value	EFS	P value	RFS	P value
Age Med: 10.7 year	>11 year	44	88.6%	0.82	72.7%	0.97	77.3%	0.78
	<11 year	45	82.2%		73.3%		86.7%	
Sex M:F: 1.8:1	Female	31 (34.8%)	80.6%	0.32	77.4%	0.62	93.5%	0.07
	Male	58 (65.2%)	87.9%		70.7%		75.9%	
ALK Status	Positive	76 (85.4%)	86.8%	0.39	75%	0.42	82.9%	0.67
	Negative	13 (14.6%)	76.9%		61.5%		76.9%	
Stage	Stage I	7 (7.8%)	100%	0.39	85.7%	0.06	85.7%	0.01
	Stage II	21 (22.4%)	85.7%		81%		95.2%	
	Stage III	44 (49.4%)	86.4%		79.5%		86.4%	
	Stage IV	17 (19.1%)	76.5%		41.2%		52.9%	
B Symptoms	Yes	23 (25.8%)	82.6%	0.4	60.9%	0.09	69.9%	0.04
	No	66 (74.2%)	86.4%		77.3%		86.4%	
BM Involvement	Yes	2 (2.2%)	50%	0.19	50%	0.56	50%	0.30
	No	87 (97.8%)	82.8%		73.6%		82.8%	
Skin Involvement	Yes	9 (10.1%)	77.8%	0.3	55.6%	0.17	55.6%	0.02
	No	80 (89.9%)	86.3%		75%		85%	
Bone Involvement	Yes	10 (11.2%)	90%	0.62	70%	0.98	70%	0.45
	No	79 (88.8%)	84.8%		73.4%		83.5%	
Visceral Involvement	Yes	9 (10.1%)	77.8%	0.39	33.3%	0.001	33.3%	0.0001
	No	80 (89.9%)	86.3%		77.5%		87.5%	
CNS Involvement	Yes	9 (10.1%)	77.8%	0.24	22.2%	0.03	44.4%	0.0001
	NO	80 (89.9%)	86.3%		78.8%		86.3%	
CD3 Status	Positive	31 (34.8%)	90.3%	0.39	74.2%	0.91	80.6%	0.78
	Negative	58 (65.2%)	82.8%		72.4%		82.8%	

Furthermore, there was a significant difference in EFS and RFS based on visceral and CNS involvement, with lower rates in individuals with visceral or CNS involvement compared to those without (**Table 2**).

### CD3 status

Univariant analysis showed no significant difference in OS, EFS, or RFS according to CD3 status (positive vs negative) (**Table 2**). There were no statistically significant association between CD3 status and other patient characteristics including age, sex, ALK status, stage, B symptoms, BM involvement, skin involvement, bone involvement, and visceral involvement. However, the relationship between CD3-positive cases and CNS involvement was statistically significant (p = 0.03).

## Discussion

Survival of ALCL has been improved over the last decade. The addition of Brentuximab resulted in a 2-year event-free survival (EFS) was 79.1%. and 76.8% with the addition of Crizotinib OS was 95-97% (8, 9).

There has been a desire to identify biological risk factors to predict outcome and tailor upfront therapy. CD3 expression is one of the risk factors that has been identified although its impact has not been completely defined.

In this study, we looked at the characteristics and survival rates of 89 patients with ALCL who were treated at Children Cancer Hospital Egypt.

The median age of 10.7 years and the predominance of male gender and ALK positivity in the study are consistent with previous reports (10, 11).

The most common stage at diagnosis in our population was stage III (49.4%), followed by stage IV (19.1%). Thus the majority of our patients had advanced disease upon presentation, which could imply a delay in diagnosis or a lack of access to early detection and treatment. Previous investigations have found varying frequencies of advanced-stage ALCL, ranging from 25% to 60% (9). The prevalence of B symptoms in our sample was 25.8%, which is lower than the frequency reported in some studies (up to 50%) (11) but greater than the frequency reported in others (around 10%) (12). This may be due to discrepancies in the definition and assessment of B symptoms.

We investigated how numerous factors affected survival. We demonstrate a 5-year OS, EFS, and RFS rates for the entire group of 84.3%, 73.1%, and 81.5%, respectively. These percentages are consistent with those reported in previous investigations of pediatric and adolescent ALCL patients (10). In our study, there was no significant difference in OS, EFS, or RFS based on age, gender, ALK status, or CD3 status. These findings are in contradiction to some recent studies finding a better prognosis in younger patients (13), female patients (13), ALK-positive patients, and CD3-negative patients (14). However, some investigations have found no significant correlation between these parameters and survival outcomes (10). The disparities between studies could be attributed to changes in sample size, patient selection, treatment regimens, follow-up duration, and statistical methodologies.

There was a substantial difference in RFS depending on stage, B symptoms, and skin involvement. Patients with stage I or II disease lacking B symptoms and skin involvement had a longer RFS than those with stage III or IV disease, B symptoms, and skin involvement. These findings are consistent with prior research, which identified advanced-stage disease (11), B symptoms (9), and skin involvement (11) as poor prognostic factors.

Furthermore, we discovered a substantial variation in EFS and RFS based on visceral and CNS involvement. Patients with visceral or CNS involvement exhibited poorer EFS and RFS compared to those without. These findings are consistent with earlier research suggesting an inferior prognosis for patients with visceral or CNS involvement (11). This extranodal involvement may indicate a more aggressive biology that is less susceptible to traditional treatment.

In our study only 9 patients had CNS involvement, 6 of which had CD3 positive tumors. Prior studies have linked CD3 expression with outcome. The high incidence of CD3 positive tumors among patients with CNS involvement in our study may support these previous studies. However, the results have to be interpreted with caution because of the small number of patients.

Our study has several limitations, which should be noted. First, it is a retrospective study using data from a single center, which may restrict its generalizability and add potential biases. Second, the small sample size may limit the reproducibility of the findings. Therefore, further work in a larger cohort is needed to investigate the of CD3 positivity in relation to the occurrence of CNS involvement in pediatric ALCL.

## Conclusion

This study indicates that CD3 expression may not be a major factor predicting survival in newly diagnosed pediatric ALCL. Additional research is needed to understand its association with CNS disease.

## Data Availability

The raw data supporting the conclusions of this article will be made available by the authors, without undue reservation.
