# Corrigendum: Impact of CD3 expression on outcome in pediatric anaplastic large cell lymphoma

**DOI:** 10.3389/fonc.2025.1630885

**Published:** 2025-06-12

**Authors:** Ahmed Salah, Hanafy Hafez, Samah Semary, Eman Naguib Khorshed, Sonia Soliman, Iman Zaky, Shahenda M. Shahin, Leslie Lehmann, Alaa ElHaddad

**Affiliations:** ^1^ Department of Pediatric Oncology, Children’s Cancer Hospital (57357), Cairo, Egypt; ^2^ Department of Pediatric Oncology, National Cancer Institute, Cairo, Egypt; ^3^ Department of Clinical Oncology, Faculty of Medicine, Beni-Suef University, Beni-Suef, Egypt; ^4^ Department of Surgical Pathology, Children’s Cancer Hospital (57357), Cairo, Egypt; ^5^ Department of Surgical Pathology, National Cancer Institute (NCI), Cairo University, Cairo, Egypt; ^6^ Department of Clinical Pathology, Children’s Cancer Hospital (57357), Cairo, Egypt; ^7^ Department of Clinical Pathology, National Cancer Institute (NCI), Cairo University, Cairo, Egypt; ^8^ Department of Radio-diagnosis, Children’s Cancer Hospital (57357), Cairo, Egypt; ^9^ Department of Clinical Research, Children’s Cancer Hospital (57357), Cairo, Egypt; ^10^ Department of Pediatric Oncology, Dana- Farber Cancer Institute, Boston, MA, United States

**Keywords:** CD3, Survival, Pediatric Anaplastic Large Cell Lymphoma, ALK, Outcome

In the published article, an author name was incorrectly written as “Eman Nageb”. The correct spelling is “Eman Naguib” Khorshed.

In the published article, **Supplementary Figures** were mistakenly not included in the publication. The missing **Supplementary Figures** have been uploaded.

The authors apologize for these errors and state that this does not change the scientific conclusions of the article in any way. The original article has been updated.

