# Supplementary material for: Impact of CD3 expression on outcome in pediatric anaplastic large cell lymphoma
Source: Front Oncol. 2025 May 15;15:1569370. doi: 10.3389/fonc.2025.1569370 (PMC12119632; doi:10.3389/fonc.2025.1569370)
Supplement: Supplementary file 1 [file DataSheet1.docx]

| 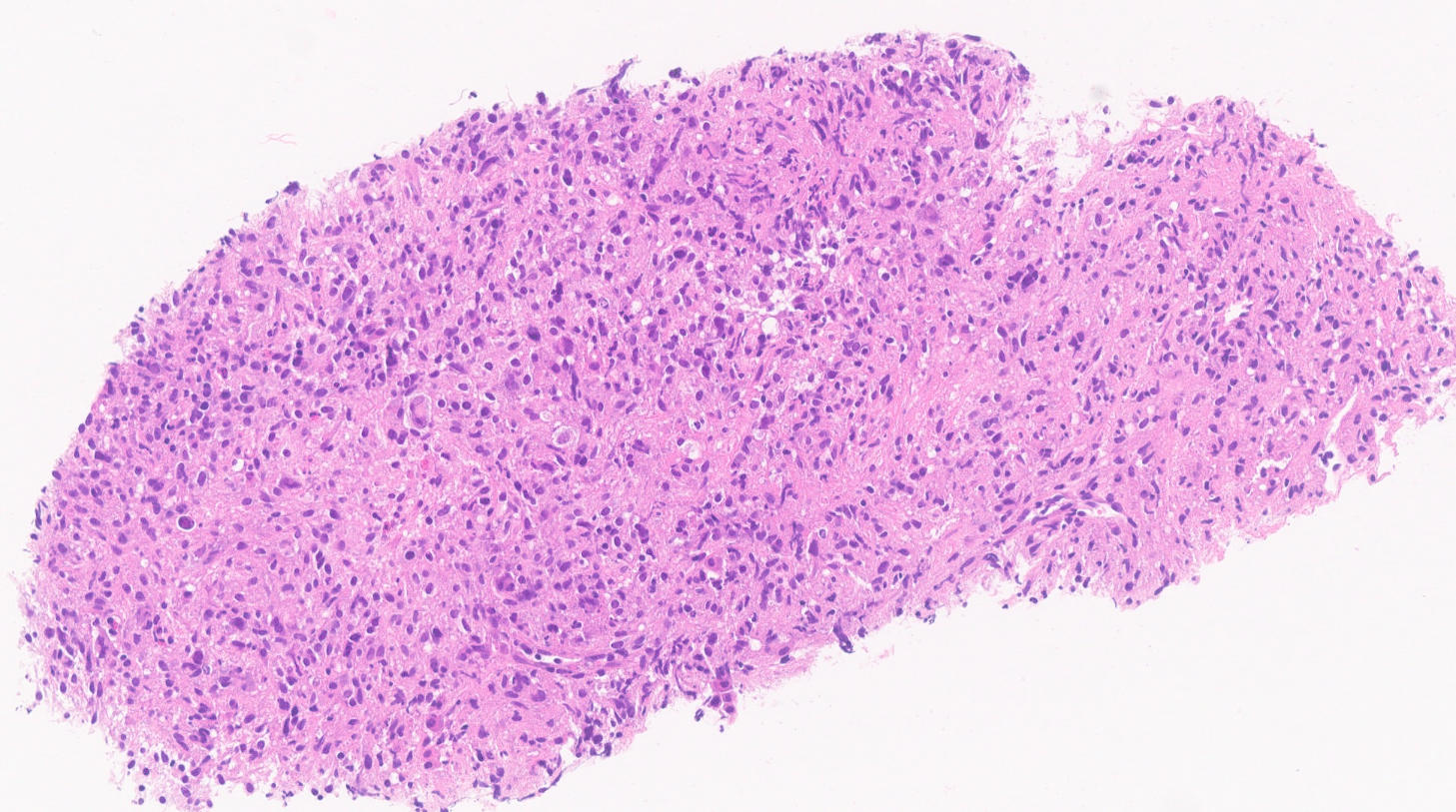  **A** |
| --- |
| 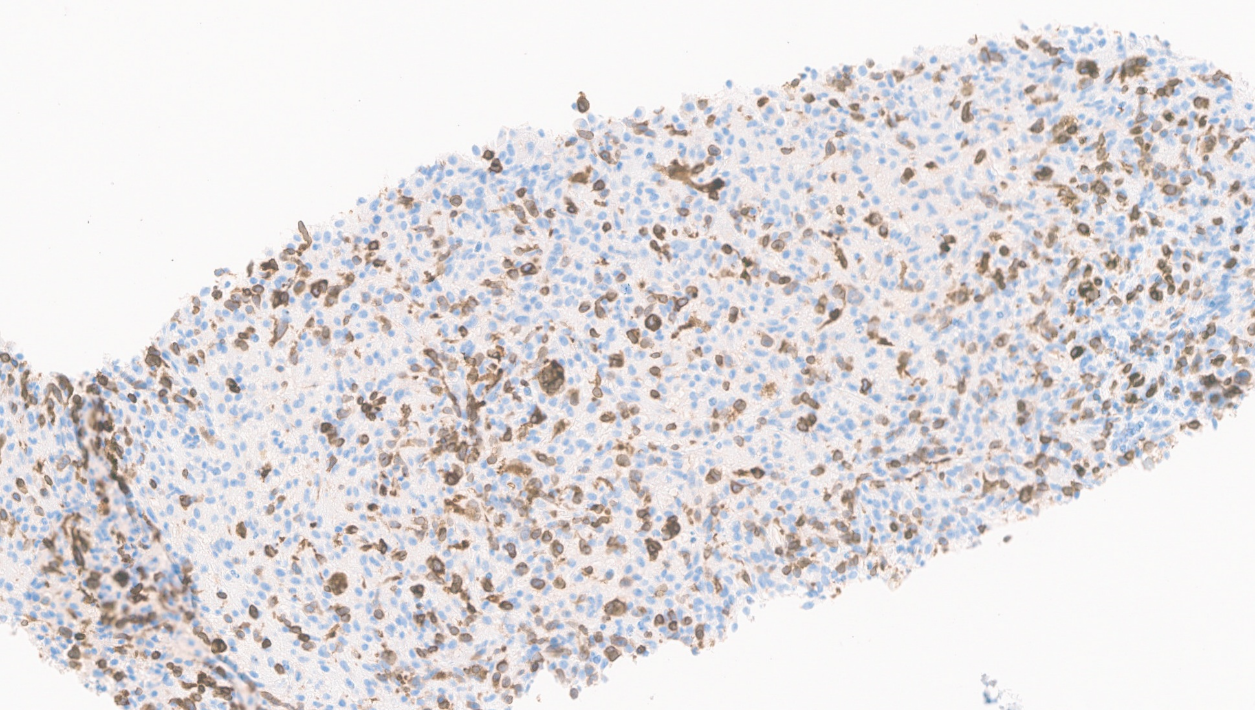  **B** |

Figure (1): A case of anaplastic large cell lymphoma. (A) Polymorphic infiltrate with scattered large neoplastic cells, H&E stain X20. (B) CD3 immunohistochemistry shows positive cytoplasmic reaction in large neoplastic cells as well as scattered reactive small T lymphocytes, IHC X20.

| 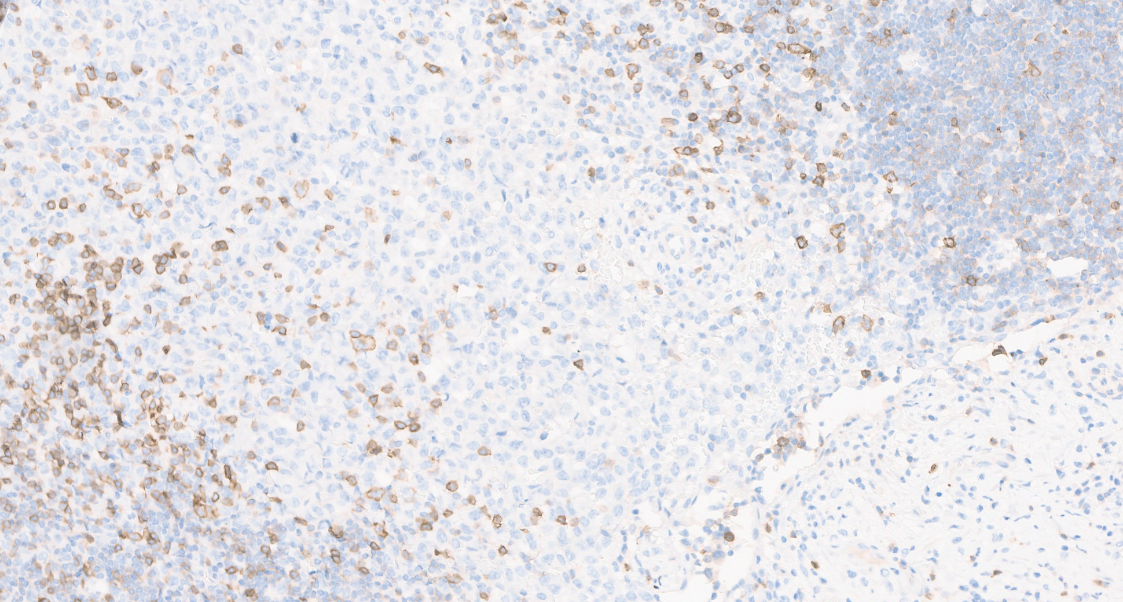  **A** |
| --- |
| **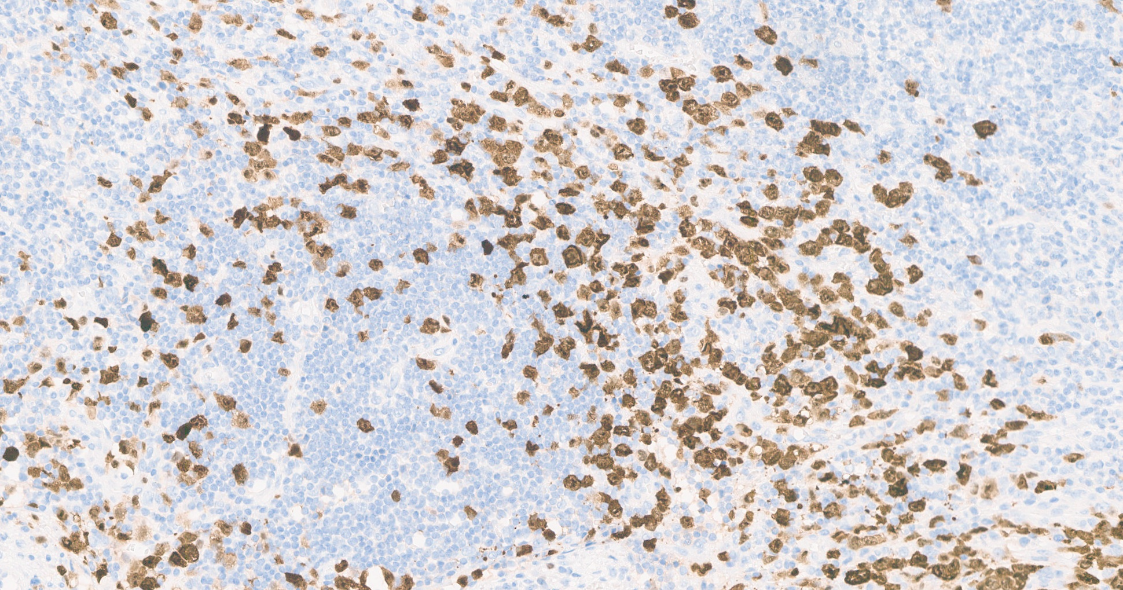**  **B** |
| 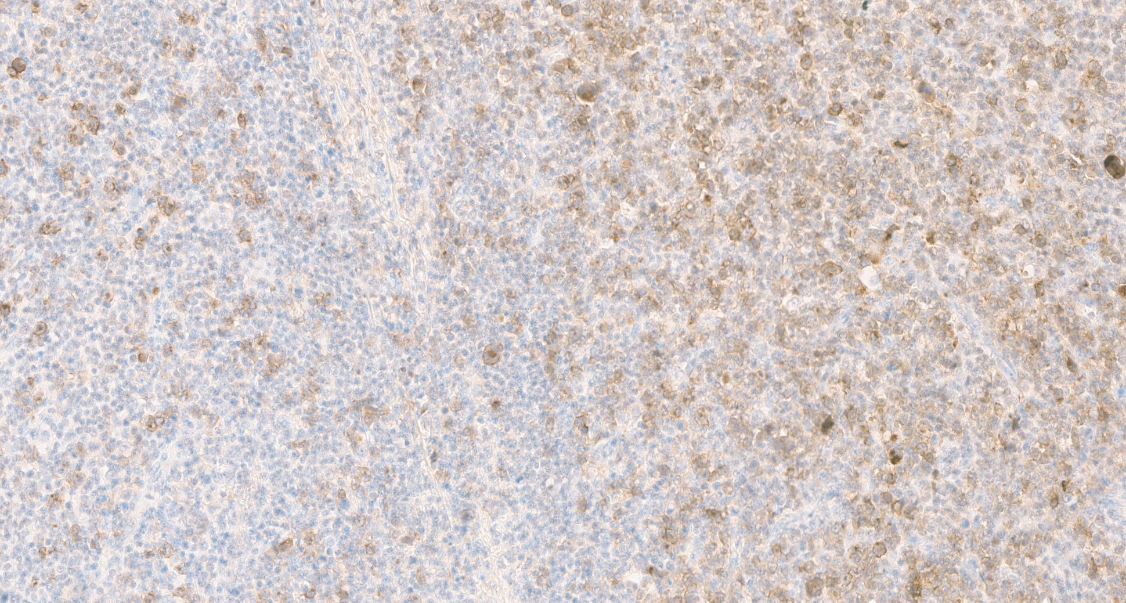  **C** |

Figure )2): A case of anaplastic large cell lymphoma. (A) CD3 immunohistochemistry shows positive cytoplasmic and membranous reaction in scattered large neoplastic cells as well as scattered reactive small T lymphocytes, IHC X20. (B) and (C) Tumor cells are positive for ALK and CD30, IHC X20.
