# Supplementary figures and images for: Impact of CD3 expression on outcome in pediatric anaplastic large cell lymphoma
Source: Front Oncol. 2025 May 15;15:1569370. doi: 10.3389/fonc.2025.1569370 (PMC12119632; doi:10.3389/fonc.2025.1569370)

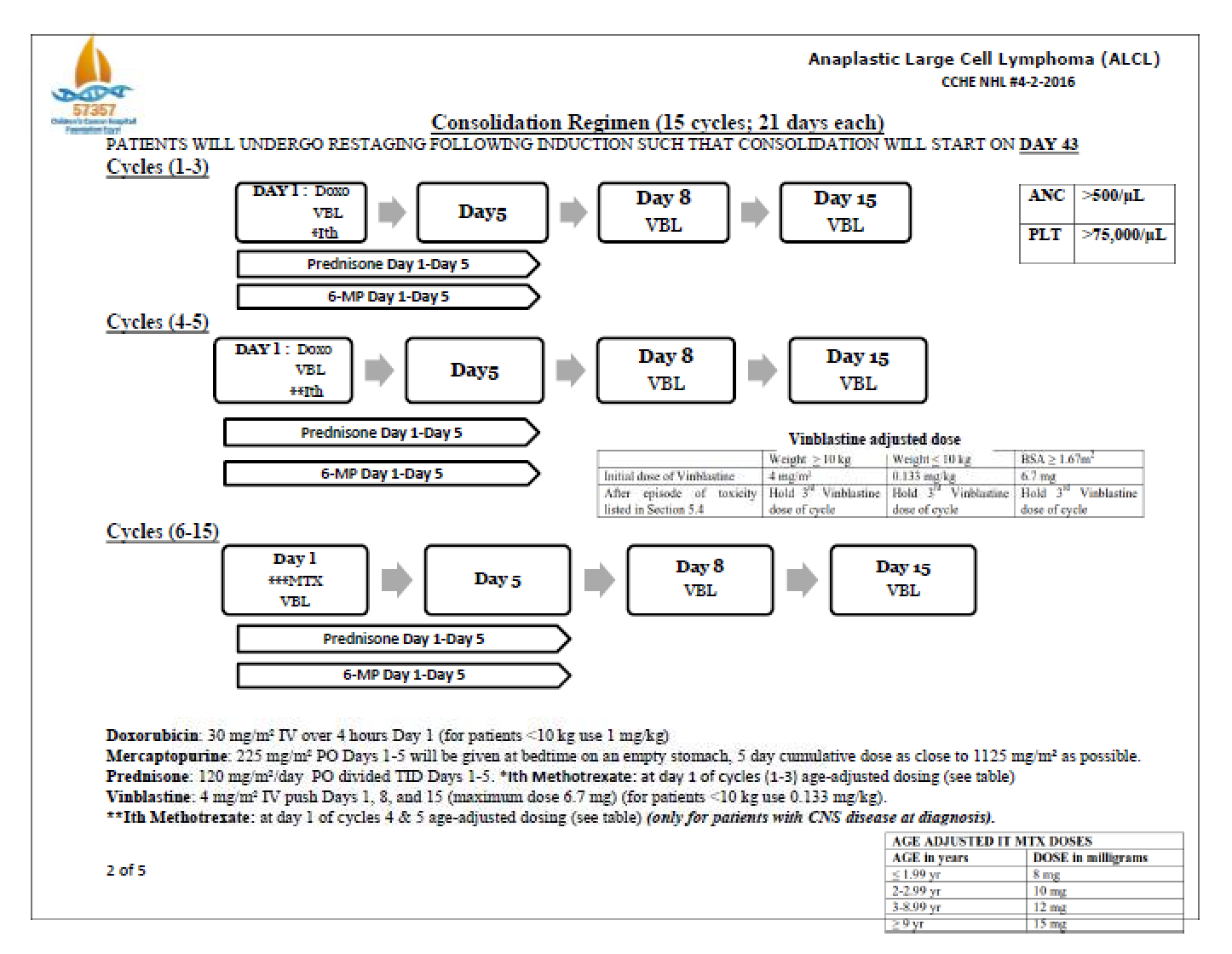

Supplement: Supplementary file 2 [file Image1.png]

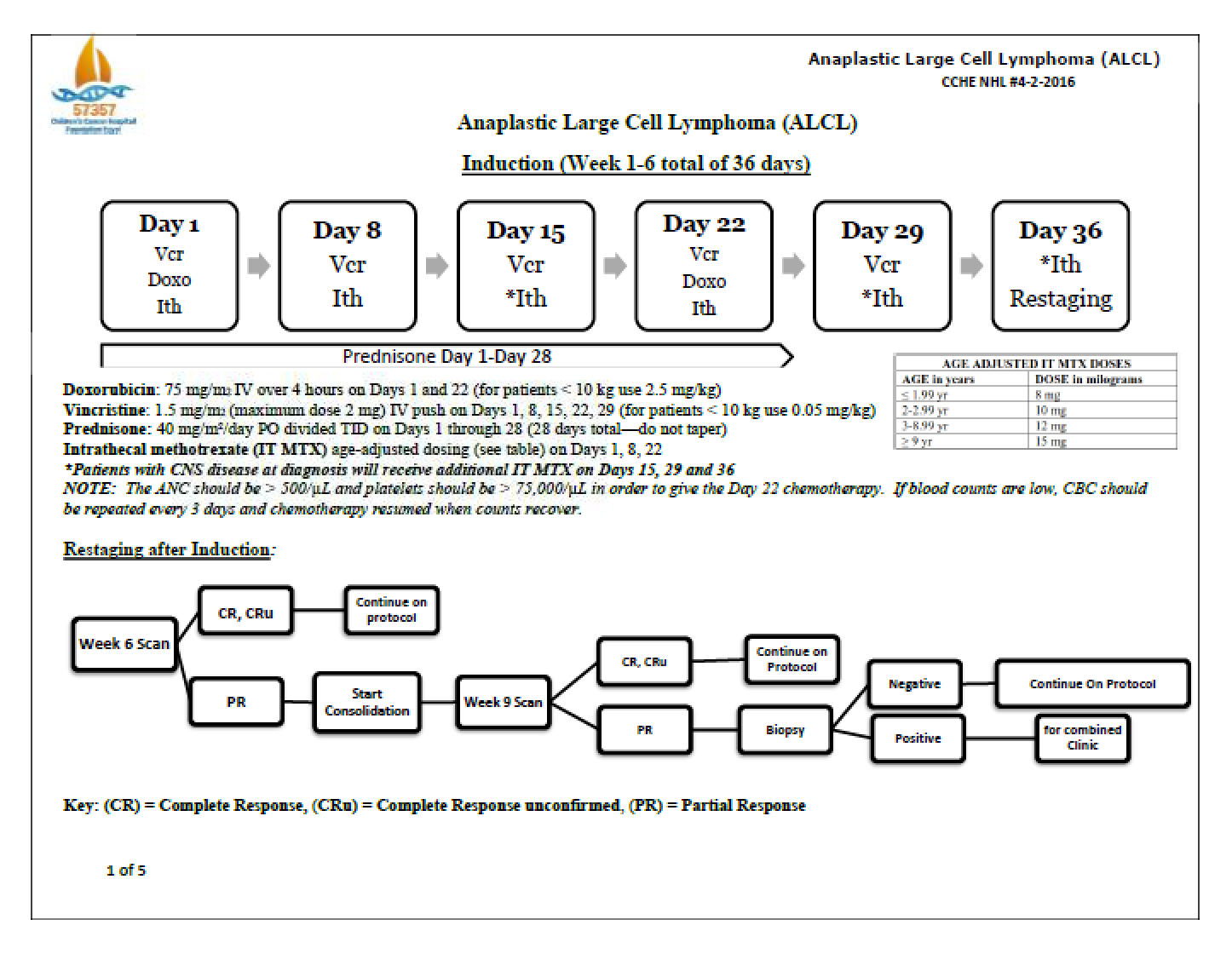

Supplement: Supplementary file 3 [file Image2.png]
